# Amino acid residues 201-205 in C-terminal acidic tail region plays a crucial role in antibacterial activity of HMGB1

**DOI:** 10.1186/1423-0127-16-83

**Published:** 2009-09-14

**Authors:** Wei Gong, Yuan Li, Fan Chao, Gang Huang, Fengtian He

**Affiliations:** 1 Department of Biochemistry and Molecular Biology, College of Basic Medical Sciences, Third Military Medical University, Chongqing, PR China

## Abstract

**Background:**

Antibacterial activity is a novel function of high-mobility group box 1 (HMGB1). However, the functional site for this new effect is presently unknown.

**Methods and Results:**

In this study, recombinant human HMGB1 A box and B box (rHMGB1 A box, rHMGB1 B box), recombinant human HMGB1 (rHMGB1) and the truncated C-terminal acidic tail mutant (tHMGB1) were prepared by the prokaryotic expression system. The C-terminal acidic tail (C peptide) was synthesized, which was composed of 30 amino acid residues. Antibacterial assays showed that both the full length rHMGB1 and the synthetic C peptide alone could efficiently inhibit bacteria proliferation, but rHMGB1 A box and B box, and tHMGB1 lacking the C-terminal acidic tail had no antibacterial function. These results suggest that C-terminal acidic tail is the key region for the antibacterial activity of HMGB1. Furthermore, we prepared eleven different deleted mutants lacking several amino acid residues in C-terminal acidic tail of HMGB1. Antibacterial assays of these mutants demonstrate that the amino acid residues 201-205 in C-terminal acidic tail region is the core functional site for the antibacterial activity of the molecule.

**Conclusion:**

In sum, these results define the key region and the crucial site in HMGB1 for its antibacterial function, which is helpful to illustrating the antibacterial mechanisms of HMGB1.

## Background

High-mobility group box 1(HMGB1) belongs to high-mobility group superfamily named for its characteristic rapid mobility in polyacrylamide gel electrophoresis (PAGE) [1]. It is highly conserved across species and wildly distribute in eukaryotic cells from yeast to human [2-4]. Structurally, human HMGB1 has 215 amino acid residues, including three main functional domains (A box, B box and a highly conservation, repetitive C terminus). The C terminus is also called C-terminal acidic tail because it is full of acidic amino acid residues such as aspartate and glutamic acid [5-8].

HMGB1 is an endogenous nuclear protein that can be translocated to the cytoplasm and then released extracellularly. In the nucleus, HMGB1 binds to DNA which functions as a co-factor critical for proper transcriptional regulation and DNA repair [9-14]. Cytoplasmal and extracellular HMGB1 has been extensively related to the growth of neurite, malignant tumor metastasis, artherosclerosis and vascellum restenosis [15-23]. HMGB1 is also detected in serum of patients with sepsis. Thus, it has been identified as a late mediator of endotoxin lethality, which plays a critical role in the processes of many kinds of acute and chronic inflammation[24,25]. Recently it is reported that the cytoplasmal and extracellular HMGB1 can act as an antibacterial factor, which is a novel function of the molecule[26,27]. In physiological situation, as a potent antibacterial protein, HMGB1 is an important part of innate immunity defensive barrier of the human body that can resist bacterial infection *in vivo*. However, the functional site for this new effect is presently unknown.

The goal of this study was to determine which region is mainly responsible for the antibacterial activity of HMGB1. We showed that the recombinant human HMGB1 (rHMGB1) and the synthetic C-terminal acidic tail (C peptide) presented antibacterial activity, nevertheless, the A box, B box domains of the molecule and the truncated HMGB1 lacking its C-terminal acidic tail failed to inhibit bacterial multiplication, which demonstrated that the C-terminal acidic tail is the key region for the antibacterial activity of HMGB1. We further showed that the amino acid residues 201-205 in C-terminal acidic tail region play a crucial role in antibacterial function of HMGB1. Therefore, our study defines the key region and the crucial site in HMGB1 for its antibacterial activity, which may provide important insights to antibacterial mechanisms of the molecule.

## Materials and methods

### Preparation of recombinant proteins and C peptide

**Figure 1:**
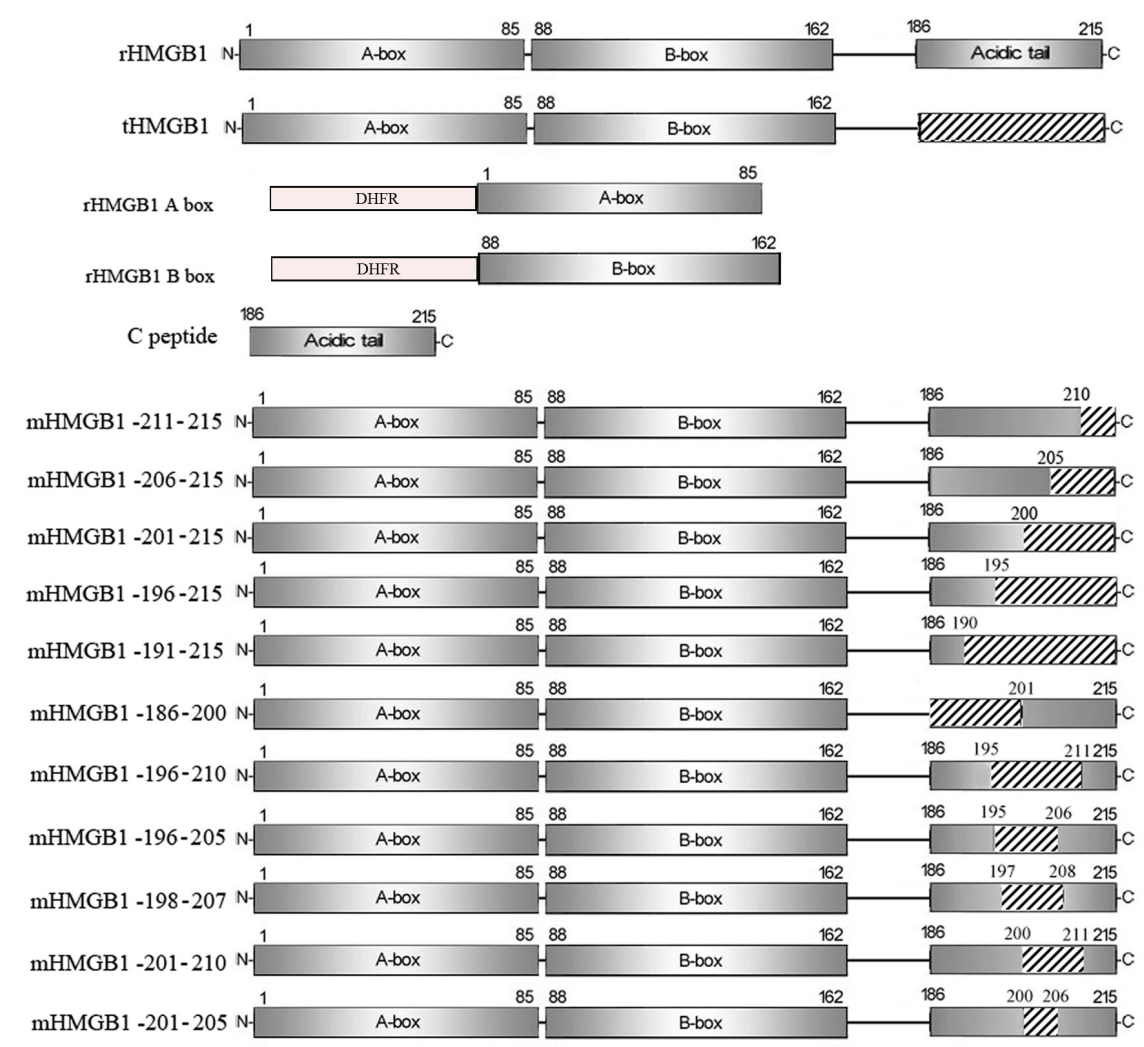
**Schematic descriptions of the structure of the recombinant proteins and C peptide used in this work**. Wild type human HMGB1 is composed of three domains: A box (amino acid residues 1-85), B boxes (amino acid residues 88-162) and a C-terminal acidic tail (amino acid residues 186-215). rHMGB1 is the full length recombinant human HMGB1. tHMGB1 is truncated HMGB1 lacking its C-terminal acidic tail. rHMGB1 A box or rHMGB1 B box is HMGB1 A box or B box fusioned with DHFR in order to stabilized express in prokaryotic system. C peptide is the C-terminal acidic tail composed of 30 amino acid residues. Eleven different deleted mutants lacking several amino acid residues in C-terminal acidic tail of HMGB1 are mHMGB1 -211-215, mHMGB1 - 206-215, mHMGB1 -201-215, mHMGB1 -196-215, mHMGB1 -191-215, mHMGB1 -186-200, mHMGB1 -196-210, mHMGB1 -196-205, mHMGB1 -198-207, mHMGB1 -201-210, and mHMGB1 -201-205. These mutants are recombinant human HMGB1 lacking amino acid residues 211-215, 206-215, 201-215, 196-215, 191-215, 186-200, 196-210, 196-205, 198-207, 201-210 and 201-205 respectively. The sprit shadow stands for the deleted regions.

A diagram of recombinant proteins and C peptide used in this work was shown in Figure 1. The cDNA encoding rHMGB1 was cloned by reverse transcription-polymerase chain reaction (RT-PCR) with total RNA extracted by TRIZOL reagent (Invitrogen) from human peripheral blood mononuclear cell (PBMC) [22,28]. Then the purified PCR product was cloned into pUC19, and the recombinant plasmid was named pUC19-rHMGB1. The DNA sequences encoding rHMGB1 A box, B box, tHMGB1 and mHMGB1 -211-215, mHMGB1 - 206-215, mHMGB1 -201-215, mHMGB1 -196-215, mHMGB1 -191-215 (rHMGB1 lacking amino acid residues 211-215, 206-215, 201-215, 196-215 and 191-215 respectively) were generated by PCR taking pUC19-rHMGB1 as template. The DNA sequences encoding mHMGB1 -186-200, mHMGB1 -196-210, mHMGB1 -196-205, mHMGB1 -198-207, mHMGB1 -201-210 and mHMGB1 -201-205 (rHMGB1 lacking amino acid residues 186-200, 196-210, 196-205, 198-207, 201-210 and 201-205 respectively) were amplified by one-step opposite-direction PCR using MutanBEST Kit (TaKaRa) according to the manufacturer's instructions (taking pUC19-rHMGB1 as template). The primers used in this study were listed in Table 1. After being sequenced, the fragments encoding rHMGB1 A box and B box were separately ligated into the *Kpn *I/*Hin*dIII cloning sites in the pQE-80L/DHFR prokaryotic expression vector (pQE-80L/DHFR vector constructed by us from pQE-80L). Other fragments were cloned into the *Kpn *I/*Hin*dIII sites of pQE-80L vector. Then, all of these prokaryotic expression vectors were separately transformed into *E. coli *DH5α and induced by IPTG to express the corresponding His-tagged proteins, which were determined by Western blot. Briefly, the lysates of *E. coli *DH5α expressing the corresponding proteins were subjected to SDS-PAGE and then transferred onto polyvinylidene difluoride membranes (Pierce). After blocked with 5% non-fat milk in phosphate buffered saline containing 0.1% Tween 20 (PBS-T), the membranes were incubated with mouse anti-His monoclonal antibody (Qiagen). Then washed with PBS-T, the membranes were incubated with horseradish peroxidase-labeled goat anti-mouse IgG (Pierce). After washed the immunoreactive proteins were visualized by DAB. The expressed proteins were purified by Ni^2+^-NTA chromatography kit (Qiagen) under the natural condition following the instructions of the manufacturer. The C peptide was synthesized by SciLight Biotechnology, LLC.

**Table 1:** Primers used for PCR.

	Forward primer	Reverse primer
rHMGB1	5'-CTGGTACCATGG	5'-CGCAAGCTTTTATGTC
A box	GCAAAGGAGATC-3'	TCCCCTTTGGGAGG-3'
rHMGB1	5'-CTGGTACCATGA	5'-CGCAAGCTTTTAATA
B box	AGTTCAAGGATCCC-3'	TGCAGCAATATCCT-3'
rHMGB1	5'-CTGGTACCATGG	5'-CGCAAGCTTTTATT
	GCAAAGGAGATC-3'	CATCATCATCATC-3'
tHMGB1	5'-CTGGTACCATGG	5'-CGCAAGCTTTTACTT
	GCAAAGGAGATC-3'	CTTTTTCTTGCT-3'
mHMGB1	5'-CTGGTACCATGG	5'-CGCAAGCTTTTATTC
-211~215	GCAAAGGAGATC-3'	TTCTTCATCTTC-3'
mHMGB1	5'-CTGGTACCATGG	5'-CGCAAGCTTTTAATCTT
-206~215	GCAAAGGAGATC-3'	CTTCATCTTCC-3'
mHMGB1	5'-CTGGTACCATGG	5'-CGAAGCTTTTACTC
-201~215	GCAAAGGAGATC-3'	CTCCTCCTCATCCT-3'
mHMGB1	5'-CTGGTACCATGG	5'-GCAAGCTTTTACTCTT
-196~215	GCAAAGGAGATC-3'	CATCTTCCTCATC-3'
mHMGB1	5'-CTGGTACCATGG	5'-CGCAAGCTTTTAATC
-191~215	GCAAAGGAGATC-3'	TTCCTCCTCTTCC-3'
mHMGB1	5'-GAAGATGAAGAA	5'-CTTCTTTTTCTT
-186~200	GATGAAGATGAA-3'	GCTTTTTTCAGC-3'
mHMGB1	5'-GATGATGATG	5'-CTCTTCATCTT
-196~210	ATGAATAAAAG-3'	CCTCATCTT-3'
mHMGB1	5'-GAAGATGAAGA	5'-CTCTTCATCTT
-196-205	AGAAGATGATG-3'	CCTCATCTT-3'
mHMGB1	5'-GAAGAAGAT	5'-CTCATCCTCTTC
-198~207	GATGATGATG-3'	ATCTTCCTCAT-3'
mHMGB1	5'-GATGATGATGAT	5'-CTCCTCCTCCT
-201-210	GAATAAAAGC-3'	CATCCTCTT-3'
mHMGB1	5'-GAAGATGAAGA	5'-CTCCTCCTCCT
-201~205	AGAAGATGATG-3'	CATCCTCTT-3'

### Antibacterial assay with bacterial dilution in the test tubes

Briefly, the bacteria were grown overnight at 37°C in Luria-Bertani (LB) agar plate, and the fresh single clone of the bacteria was picked up and inoculated into LB liquid medium, shaking cultivated at 37°C until the optical density at 600 nm (OD_600_) reaching 0.4. Five microliter aliquot of the bacteria was 1:1000 diluted in each tube containing LB liquid medium, and then the tested recombinant protein or C peptide was added into different tubes at a final concentration of 0, 2, 4, 6, 8 and 10 μmol/L. After shaking cultivated at 37°C for 8 hours, the OD_600 _of the bacteria was measured using a spectrophotometer. Each experiment was performed in triplicate and repeated three times. DHFR protein purified by the same system was used as a negative control. The bacteria used in this experiment included *Staphylococcus aureus *(SA), *E. coli *JM109 (JM109), *E. coli *ATCC 25922 (ATCC 25922), *E. coli *DH5α (DH5α) and *Pseudomonas aeruginosa *(PA).

### Antibacterial assay with dispersion method

Briefly, the double-layer LB agar plates were prepared (the upper layer containing 50 μl bacteria with OD_600 _= 0.4 and 7 g/L agar, the nether layer with agar at 15 g/L). The holes in the upper layer of the plate were made by a sterile hoop with a diameter of 5 mm. Then tested recombinant protein or C peptide was loaded into the holes (300 nmol/hole). After incubation at 37°C for 8 hours, the diameters of the zones of growth inhibition were measured as reflections of the ability of the corresponding protein or C peptide to inhibit the bacteria multiplication. DHFR protein purified by the same system was used as a negative control. The bacteria used in this experiment were same as in "*Antibacterial assay with bacterial dilution in the test tubes*".

### Statistical analysis

Data were plotted as mean ± standard deviation and normalized relative to the negative control. Comparison between data sets obtained for different recombinant proteins and peptide were performed with one-way ANOVA using software SPSS13.0.

## Results

### Preparation of recombinant proteins

**Figure 2:**
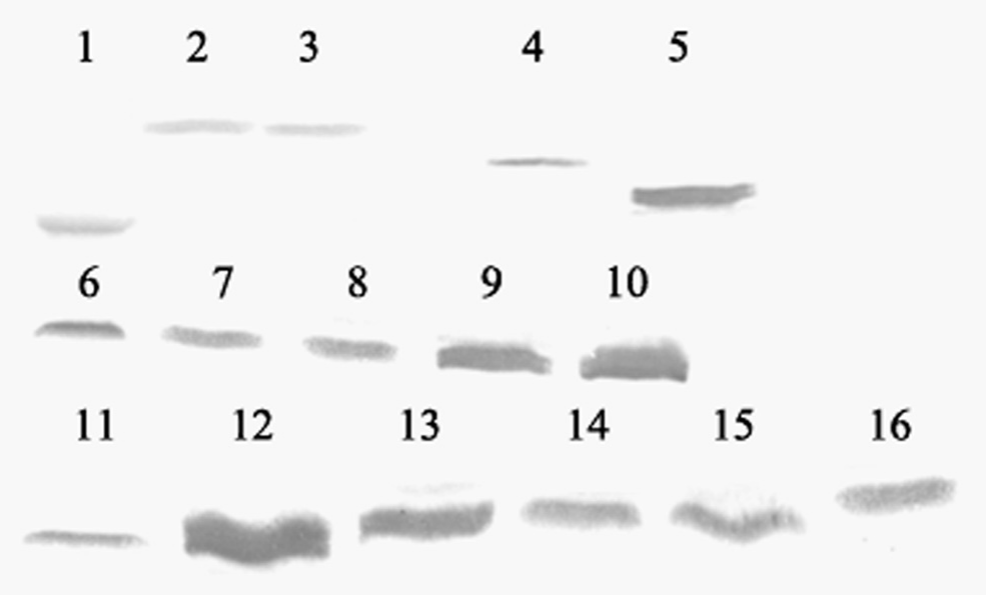
**Western blot analysis of the recombination proteins**. Identification of the expression of the His-tagged recombinant proteins in *E. Coli *DH5α by Western blot. 1, DHFR; 2, rHMGB1 A box; 3, rHMGB1 B box; 4, rHMGB1; 5, tHMGB1; 6, mHMGB1 -211-215; 7, mHMGB1- 206-215; 8, mHMGB1 -201-215; 9, mHMGB1 -196-215; 10, mHMGB1 -191-215; 11, mHMGB1 -186-200; 12, mHMGB1 -196-210; 13, mHMGB1 -196-205; 14, mHMGB1 -198-207; 15, mHMGB1 -201-210; 16, mHMGB1 -201-205.

**Figure 3:**
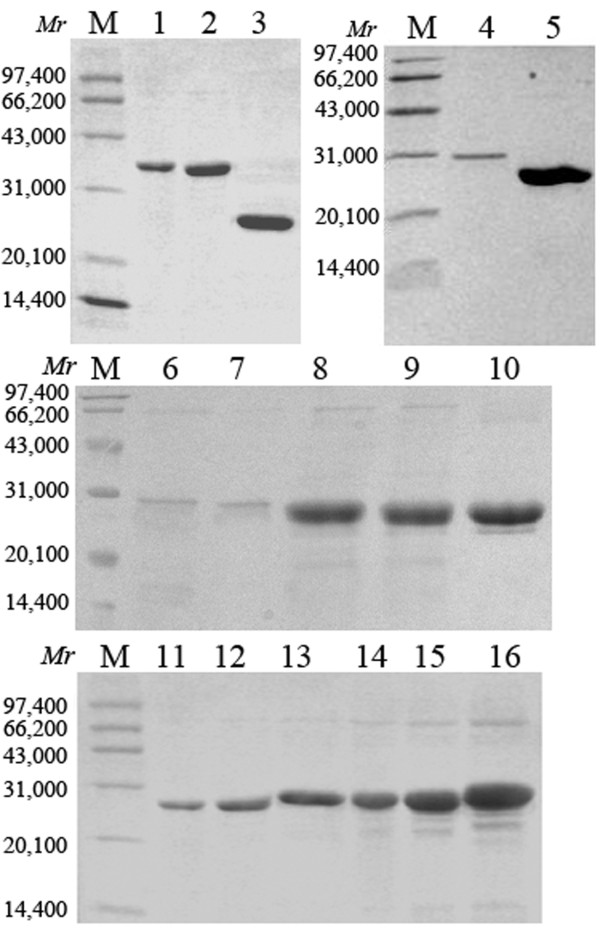
**SDS-PAGE analysis of the recombination proteins**. Purity analysis of the recombinant proteins by SDS-PAGE. After purified by Ni^2+^-NTA chromatography the purity of the proteins were analyzed with SDS-PAGE. M, Molecular weight standards; 1, rHMGB1 A box; 2, rHMGB1 B box; 3, DHFR; 4, rHMGB1; 5, tHMGB1; 6, mHMGB1 -211-215; 7, mHMGB1 -206-215; 8, mHMGB1 -201-215; 9, mHMGB1 -196-215; 10, mHMGB1 -191-215; 11, mHMGB1 -186-200; 12, mHMGB1 -196-210; 13, mHMGB1 -196-205; 14, mHMGB1 -198-207; 15, mHMGB1 -201-210; 16, mHMGB1 -201-205.

Totally 16 recombinant proteins were prepared in this study (a schematic description of the recombinant proteins was shown in Figure 1), including rHMGB1, tHMGB1, rHMGB1 A box, rHMGB1 B box, eleven different deleted mutants lacking several amino acid residues in C-terminal acidic tail of HMGB1 (rHMGB1 lacking amino acid residues 211-215, 206-215, 201-215, 196-215, 191-215, 186-200, 196-210, 196-205, 198-207, 201-210 and 201-205 respectively) and negative control protein DHFR. All the 16 proteins were 6×His-tagged because of the characteristics of the expression vector pQE-80L, which can result in a 6×His-tag added in N-terminal of the recombinant proteins. The expressed proteins by *E. coli *DH5α were confirmed by western blot with anti-His antibody (as shown in Figure 2). After purified by Ni^2+^-NTA chromatography, the purity of the proteins was analyzed by SDS-PAGE (Figure 3), which could reach 90%-95%. The C peptide was synthesized and HPLC purified by SciLight Biotechnology, LLC (Beijing, CHINA) at 96% purity.

### C-terminal acidic tail is the key region for the antibacterial activity of HMGB1

**Figure 4:**
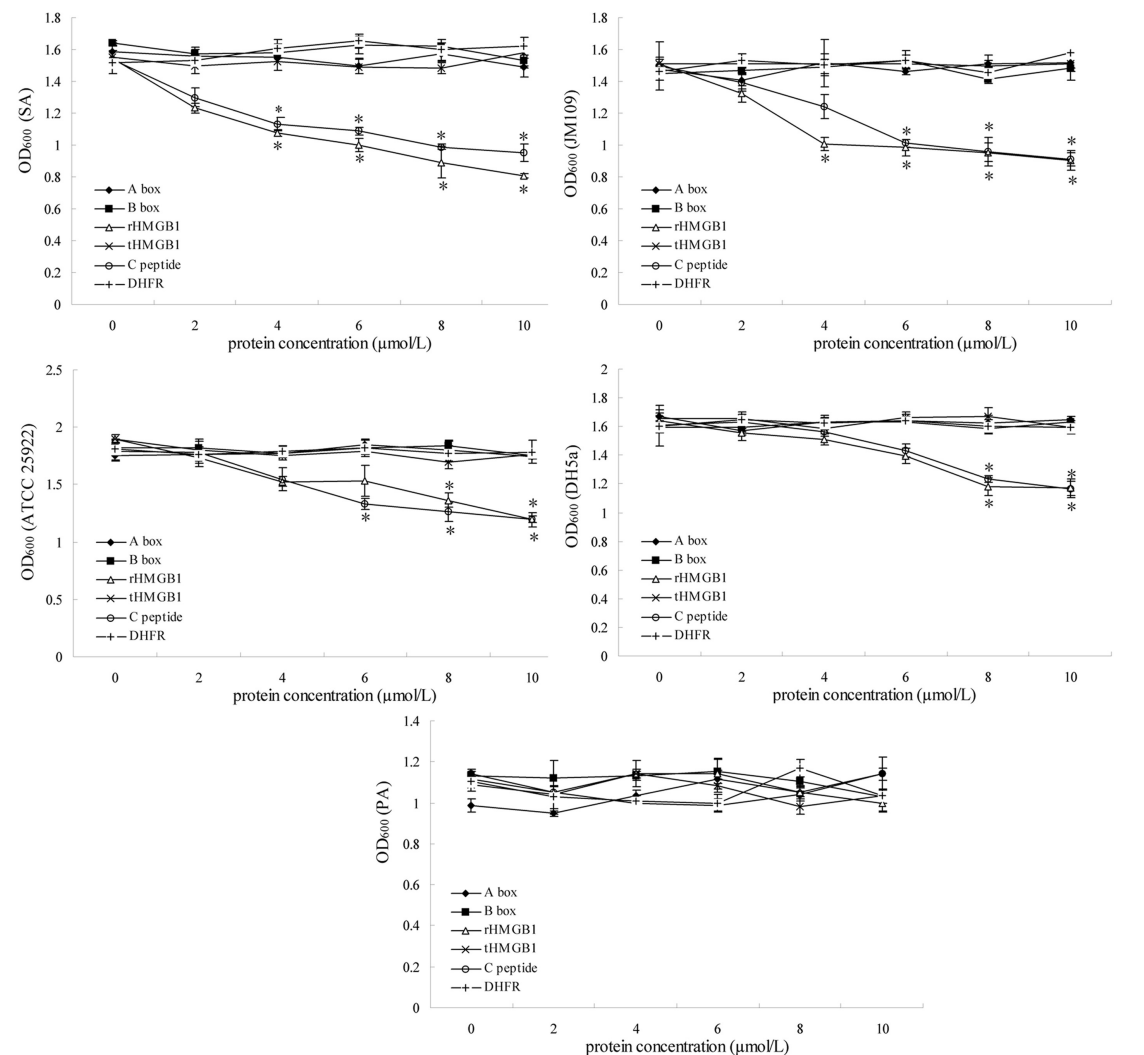
**Antibacterial activity assay of rHMGB1, tHMGB1, rHMGB1 A box, B box, and C peptide with the method of dilution in the test tubes**. The antibacterial activities of rHMGB1, tHMGB1, rHMGB1 A box, B box, and C peptide were compared by the method of dilution in the test tubes as described in the *Materials and Methods*. The bacteria used in this experiment included SA, JM109, ATCC 25922, DH5α and PA. Each experiment was performed in triplicate and repeated three times. DHFR protein purified by the same system was used as a negative control. Data showed as mean ± standard deviation and were analyzed with One-Way ANOVA. * *P *< 0.05 *vs *control DHFR.

To examine which region is mainly responsible for the antibacterial activity of HMGB1, the antibacterial functions of rHMGB1, tHMGB1, rHMGB1 A box and B box, and C peptide were compared. As shown in Figure 4 and additional file 1 panel A-F, both the full length rHMGB1 and C peptide alone could inhibit the proliferation of SA, JM109, ATCC 25922 and DH5α in different extent, but rHMGB1 A box and B box, and tHMGB1 lacking the C-terminal acidic tail had no effect on the tested bacteria, which indicated that the C-terminal acidic tail is the key region for the antibacterial activity of HMGB1. Moreover, the results also showed that all the above four proteins and the C peptide failed to inhibit PA multiplication, which suggested that the sensitivity of different bacteria to HMGB1 may be distinct.

### Amino acid residues 201-205 in C-terminal acidic tail region is the core functional site for the antibacterial activity of HMGB1

**Figure 5:**
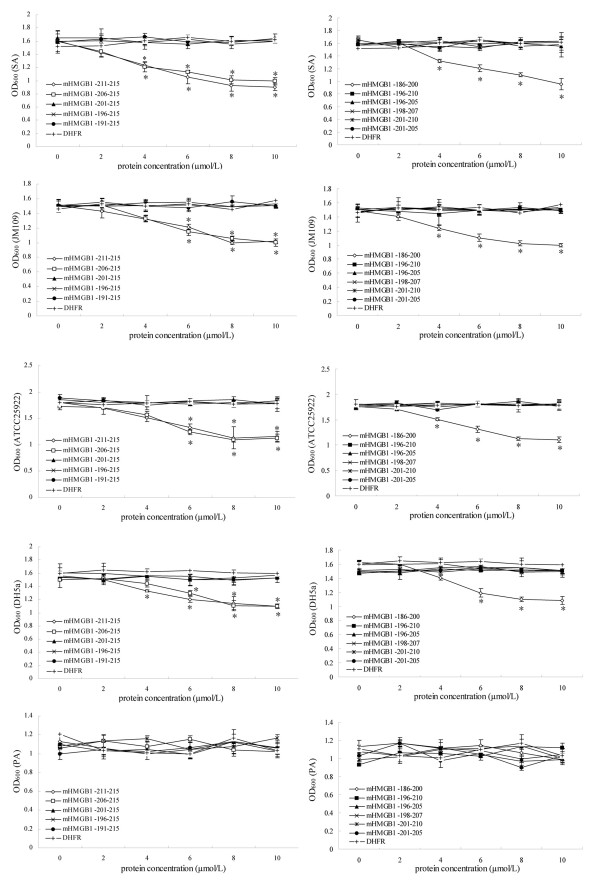
**Antibacterial activity assay of eleven different deleted mutants lacking different several amino acid residues in C-terminal acidic tail region of HMGB1 with the method of dilution in the test tubes**. The antibacterial activities of eleven different deleted mutants lacking different several amino acid residues in C-terminal acidic tail region of HMGB1 were compared by the method of dilution in the test tubes. The bacteria used in this experiment included SA, JM109, ATCC 25922, DH5α and PA. Each experiment was performed in triplicate and repeated three times. DHFR protein purified by the same system was used as a negative control. Data showed as mean ± standard deviation and were analyzed with One-Way ANOVA. * *P *< 0.05 *vs *control DHFR.

To determine which site in the C-terminal acidic tail region plays a crucial role in antibacterial activity of HMGB1, we constructed eleven different deleted mutants lacking different several amino acid residues in C-terminal acidic tail region of HMGB1 (as shown in Figure 1). The antibacterial assay with these mutants showed that three of them (lacking amino acid residues 211-215, 206-215 and 186-200 respectively) could still exert antibacterial function on SA, JM109, ATCC25922 and DH5α, but the other eight mutants failed to inhibit any one of the above bacterial multiplication (Figure 5 and additional file 1 panel F-Q). These results indicated that the amino acid residues 201-205 in C-terminal acidic tail region is the core functional site for the antibacterial activity of HMGB1, which means this site plays a crucial role in the antibacterial function of the molecule. In addition, similar with the results in "*C-terminal acidic tail is the key region for the antibacterial activity of HMGB1*", all the eleven mutants had no effect on bacteria PA multiplication.

## Discussion

We have primarily demonstrated in this study that the C-terminal acidic tail is the key region for the antibacterial activity of HMGB1, and the amino acid residues 201-205 in C-terminal acidic tail region is the core functional site for this activity of the molecule. Moreover, the sensitivity of different bacteria to HMGB1 varies greatly.

The antibacterial activity is a novel function of HMGB1. Because of the new effect the cytoplasmal and extracellular HMGB1 have been considered an important part of innate immunity defensive barrier of the human body in physiological situation. The recent study shows that the purified testicular HMGB1 has antibacterial activity, which means this protein may function as a paracrine host defense factor in the testis[26,27]. In addition, it has been reported that the recombinant rat HMGB1 expressed by prokaryotic system has similar antibacterial activity as native human HMGB1[26]. However, no information is available at present as to which region in HMGB1 is mainly responsible for its antibacterial activity. In this study we identified that the C-terminal acidic tail is the key region for the antibacterial activity of HMGB1, because both the full length rHMGB1 and C peptide alone could efficiently inhibit bacteria proliferation, but rHMGB1 A box and B box, and tHMGB1 lacking the C-terminal acidic tail had no antibacterial function. Furthermore, we identified that the amino acid residues 201-205 in C-terminal acidic tail region may play a crucial role in the antibacterial activity of HMGB1. Our finding would be of helpful to define the antibacterial mechanisms of HMGB1.

Because of the antibacterial activity it is difficult to express high level HMGB1 with prokaryotic system. Previous studies have shown the difficulties in expressing the molecule in *E. coli*, indicating that the choice of expressing vector is crucial for the yield of recombinant HMGB1[24,29]. It is reported that pQE-80L is suitable for expressing toxic protein because there is no leaky expression before IPTG inducing. Therefore, this vector was used in this study. Although we can get enough rHMGB1 for the *in vitro *experiment, the expression level is still low no matter how the expressional conditions were optimized. However the proteins without antibacterial activity could be highly expressed. Our results is consistent with the previously reports[24,29]. This phenomenon suggests that it is necessary and important to develop novel expression system for preparing abundant rHMGB1 for the *in vivo *experiment. Moreover, as mentioned below, *E. coli *DH5α has the weakest sensitivity to HMGB1, which suggest that besides the antibacterial activity of HMGB1 there may be other reasons responsible for the low level expression of the molecule.

Different bacteria may have a varied sensitivity to HMGB1, so we carefully chose experimental bacteria strains in this study. SA is a typical gram-positive cocci genus[30]. *E. coli *is a large group of gram-negative rode whose natural habitat is the intestinal tract. *E. coli *JM109 and DH5α are widely used in gene engineering today. *E. coli *ATCC 25922 is the standard stain of *E. coli*. PA is a medically important kind of gram-negative genus[31]. However, PA is resistant to all tested proteins. At present we have no idea about the reasons why the antibacterial effect of HMGB1 on different bacteria is varied.

Results from this study may also suggest that it has some potential to develop antibacterial peptide based on C-terminal acidic tail. Antibacterial peptides have been proposed as novel drugs to target different bacteria, so they may have therapeutic implications for bacteria infections[32,33]. Our results showed that C-terminal acidic tail alone (C peptide) could exert antibacterial function and the amino acid residues 201-205 in C-terminal acidic tail region plays a crucial role in antibacterial function of HMGB1, which suggested that it may be possible to design novel antibacterial peptides derived from C peptide. The C-terminal acidic tail of HMGB1 is highly conservation, which composes of 30 residues entirely of aspartic acid and glutamic acid residues. The sequence of HMGB1 amino acid residues 201-205 is EDEED which are all acidic amino acid residues but no data and references for the reason why these residues, but not other C-terminal residues, are important for HMGB1 antibacterial function so far. More studies are warranted regarding the structure basis and molecular mechanism by which HMGB1 plays antibacterial function.

## Conclusion

This study suggests that the C-terminal acidic tail is the key region for the antibacterial activity of HMGB1, and the amino acid residues 201-205 in C-terminal acidic tail region is the core functional site for this activity of HMGB1.

## List of Abbreviations

HMGB1: High-mobility group box 1; rHMGB1: recombinant human HMGB1; tHMGB1: truncated HMGB1 lacking its C-terminal acidic tail; mHMGB1: eleven different deleted mutants lacking several amino acid residues in C-terminal acidic tail of HMGB1; mHMGB1 -211-215, mHMGB1 - 206-215, mHMGB1 -201-215, mHMGB1 -196-215, mHMGB1 -191-215, mHMGB1 -186-200, mHMGB1 -196-210, mHMGB1 -196-205, mHMGB1 -198-207, mHMGB1 -201-210, mHMGB1 -201-205: recombinant human HMGB1 lacking amino acid residues 211-215, 206-215, 201-215, 196-215, 191-215, 186-200, 196-210, 196-205, 198-207, 201-210 and 201-205 respectively; C peptide: the synthetic C-terminal acidic tail; DHFR: dihydrofolate reductase; RT-PCR: reverse transcription-polymerase chain reaction; IPTG: Isopropyl β-D-1-Thiogalactopyranoside; PBMC: peripheral blood mononuclear cell; SA: *Staphylococcus aureus*;*E. coli: Escherichia coli*; PA: *Pseudomonas aeruginosa*

## Competing interests

The authors declare that they have no competing interests.

## Authors' contributions

WG participated in the design of the study, performed all experiments, performed the statistical analysis and drafted the manuscript. FTH designed the study, participated in the writing of the manuscript and coordinated the study. YL participated in experiments and the statistical analysis. FC and GH helped WG and YL in experiments. Both authors read and approved the final manuscript.

## Supplementary Material

Additional file 1 **Antibacterial efficiency analysis of the recombinant proteins and C peptide by dispersion method (diameter/mm)**. The antibacterial activities of the recombinant proteins and C peptide were detected by dispersion method as described in *Materials and Methods*. The numbers refers to the diameters (mm) of the antibacterial cirques showed in the table. The zone without bacterial growth reflects the potency of growth inhibition. DHFR was used as a negative control. Click here for file
