# Supplementary material for: Amino acid residues 201-205 in C-terminal acidic tail region plays a crucial role in antibacterial activity of HMGB1
Source: J Biomed Sci. 2009 Sep 14;16(1):83. doi: 10.1186/1423-0127-16-83 (PMC2754419; doi:10.1186/1423-0127-16-83)
Supplement: Additional file 1 — Antibacterial efficiency analysis of the recombinant proteins and C peptide by dispersion method (diameter/mm). The antibacterial activities of the recombinant proteins and C peptide were detected by dispersion method as described in Materials and Methods. The numbers refers to the diameters (mm) of the antibacterial cirques showed in the table. The zone without bacterial growth reflects the potency of growth inhibition. DHFR was used as a negative control. [file 1423-0127-16-83-S1.doc]

| Proteins and peptide | SA | JM109 | ATCC 25922 | DH5α | PA |
| --- | --- | --- | --- | --- | --- |
| (A)  rHMGB1  A box | － | － | － | 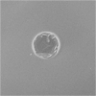  － | － |
| (B)  rHMGB1  B box | － | － | － | 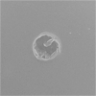  － | － |
| (C)  rHMGB1 | 14.3 | 13.9 | 12.2 | 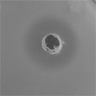  7.1 | － |
| (D)  tHMGB1 | － | － | － | － | － |
| (E)  C peptide | 10.5 | 9.2 | 8.4 | 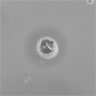  6.3 | － |
| (F)  DHFR | － | － | － | － | － |
| (G)  mHMGB1 -211-215 | 11.6 | 12.5 | 11.5 | 6.5 | － |
| (H)  mHMGB1 -206-215 | 12.8 | 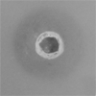  12.4 | 10.5 | 6.2 | － |
| (I)  mHMGB1 -201-215 | － | － | － | － | － |
| (J)  mHMGB1 -196-215 | 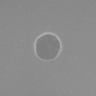  － | － | － | － | － |
| (K)  mHMGB1 -191-215 | － | － | － | － | － |
| (L)  mHMGB1 -186-200 | 12.2 | 12.7 | 10.8 | 6.4 | － |
| (M)  mHMGB1 -196-210 | － | － | － | － | － |
| (N)  mHMGB1 -196-205 | － | － | 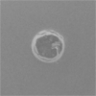  － | － | － |
| (O)  mHMGB1 -198-207 | － | － | － | － | － |
| (P)  mHMGB1 -201-210 | － | － | － | － | － |
| (Q)  mHMGB1 -201-205 | 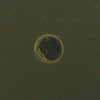 － | － | － | － | － |
